# Clinical and echocardiographic results of the MEMO 4D semi-rigid annuloplasty ring

**DOI:** 10.1186/s13019-024-02649-3

**Published:** 2024-04-01

**Authors:** Corrado Fiore, Marcello Melone, Kia Vaziri Farahani, Rebani Sinani, Anna Nicoletti, Luigi Specchia, Giuseppe Santarpino, Giuseppe Speziale

**Affiliations:** 1 Department of Cardiology, Città di Lecce Hospital, GVM Care&Research, Via Camillo Rosalba 35/37, Lecce, 70124 BA Italy; 2 Department of Anesthesiology, Città di Lecce Hospital, GVM Care&Research, Lecce, Italy; 3 Department of Cardiac Surgery, Città di Lecce Hospital, GVM Care&Research, Lecce, Italy; 4 Department of Clinical and Experimental Medicine, Magna Graecia University, Catanzaro, Italy; 5 Department of Cardiac Surgery, Department of cardiology, Paracelsus Medical University Nuremberg, Città di Lecce Hospital, GVM Care & Research, Germany, Lecce, Italy

**Keywords:** Mitral valve repair, Semirigid annuloplasty ring, Saddle shape

## Abstract

**Background:**

Mitral regurgitation is a frequent valvular disease, with an increasing prevalence. We analyzed the short-term outcomes of mitral valve repair procedures conducted in our clinic using a new semirigid annuloplasty ring featuring a gradual saddle shape design.

**Methods:**

We retrospectively analyzed mitral valve repair surgeries performed at our Institution between December 2019 and November 2021 with the MEMO 4D semirigid annuloplasty ring.

**Results:**

In total, 53 patients were included in the study. Mean patient age was 63.6 ± 11.7 years. Most patients presented with degenerative mitral valve regurgitation (*N* = 44; 83%). The grade of mitral regurgitation was equal or more than 3 + in 98.1% of the patients (*N* = 52). The most used ring size was size 34 mm (*N* = 30, 56.6%). There was no intraoperative or hospital mortality. No cases of stroke, bleeding, endocarditis or other major complications occurred. At discharge, most patients were in NYHA class I. Postoperative echocardiographic results showed no (90.6%) or 1+ (5.7%) mitral valve regurgitation. Only 1 patient (1.9%) presented with mitral valve regurgitation grade 2+. Mean postoperative transvalvular gradient was low (mean = 3.3 ± 1.2 mmHg). No cases of LVOT obstruction or systolic anterior motion occurred.

**Conclusions:**

Our series showed excellent mitral valve competency and very satisfactory early clinical outcomes. The transesophageal echocardiographic follow-up, despite obtained in a limited number of patients, further confirmed the effectiveness of findings of this preliminary experience.

## Background

Primary mitral regurgitation (MR) is characterized by the primary lesion of one or more components of the mitral valve apparatus and is usually the consequence of degenerative disease. Surgical intervention is associated with high repair rates and low operative morbidity and mortality. Therefore, current international guidelines advise mitral valve repair (MVR) for symptomatic patients with severe MR [1]. MVR with an annuloplasty ring is the gold standard technique for degenerative mitral valve insufficiency. Annuloplasty devices support the repaired leaflets and help prevent further dilation of the annulus. Designs have moved progressively toward more physiologically shaped rings and bands with less rigidity. Within the available large variety of annuloplasty rings, those that restore the physiological saddle shape have gained great interest, thanks to the reduction of the haemodynamic stress on the other valve components such as the leaflets and the chords and the optimization of leaflet coaptation [2, 3]. A saddle-shaped ring can be useful for making a flattened mitral annulus recovering the physiological shape to maintain long-term valve function [4–6]. However, mitral annulus dynamics may be strongly impeded by using a rigid or semi-rigid saddle-shaped ring, yielding limited intrinsic flexibility [7].

A “saddle-shaped” mitral annulus with an optimal ratio between annular height and commissural diameter may reduce leaflet and chordal stress and the annular height:commissural width (CW) ratio (AHCWR) has been considered in literature as a surrogate for the saddle shape of the annulus [8]. 

The Memo 4D semi-rigid annuloplasty ring (CORCYM, Saluggia, Italy) is an evolution of the MEMO 3D ring with an anterior saddle shape gradually enhanced from size 34 to size 42 as well as a progressive increase of the antero-posterior diameter in the same range of sizes.

To our knowledge there are no publications on the clinical and hemodynamic results of this new ring; therefore, we retrospectively gathered the hospital and follow-up data of patients undergoing mitral valve repair with MEMO 4D ring in our clinic.

## Patients and methods

This study is a single-centre, retrospective analysis of mitral valve repair surgeries done with the MEMO 4D ring, performed between December 2019 and November 2021. The study was approved by the site’s ethical committee and complied with the Declaration of Helsinki. Written, informed consent was not required due to the use of anonymized data already collected as part of routine practice. Demographic, preoperative, intraoperative and postoperative data were obtained from the data prospectively entered in the database of our clinic.

### Patient population

**Fig. 1:**
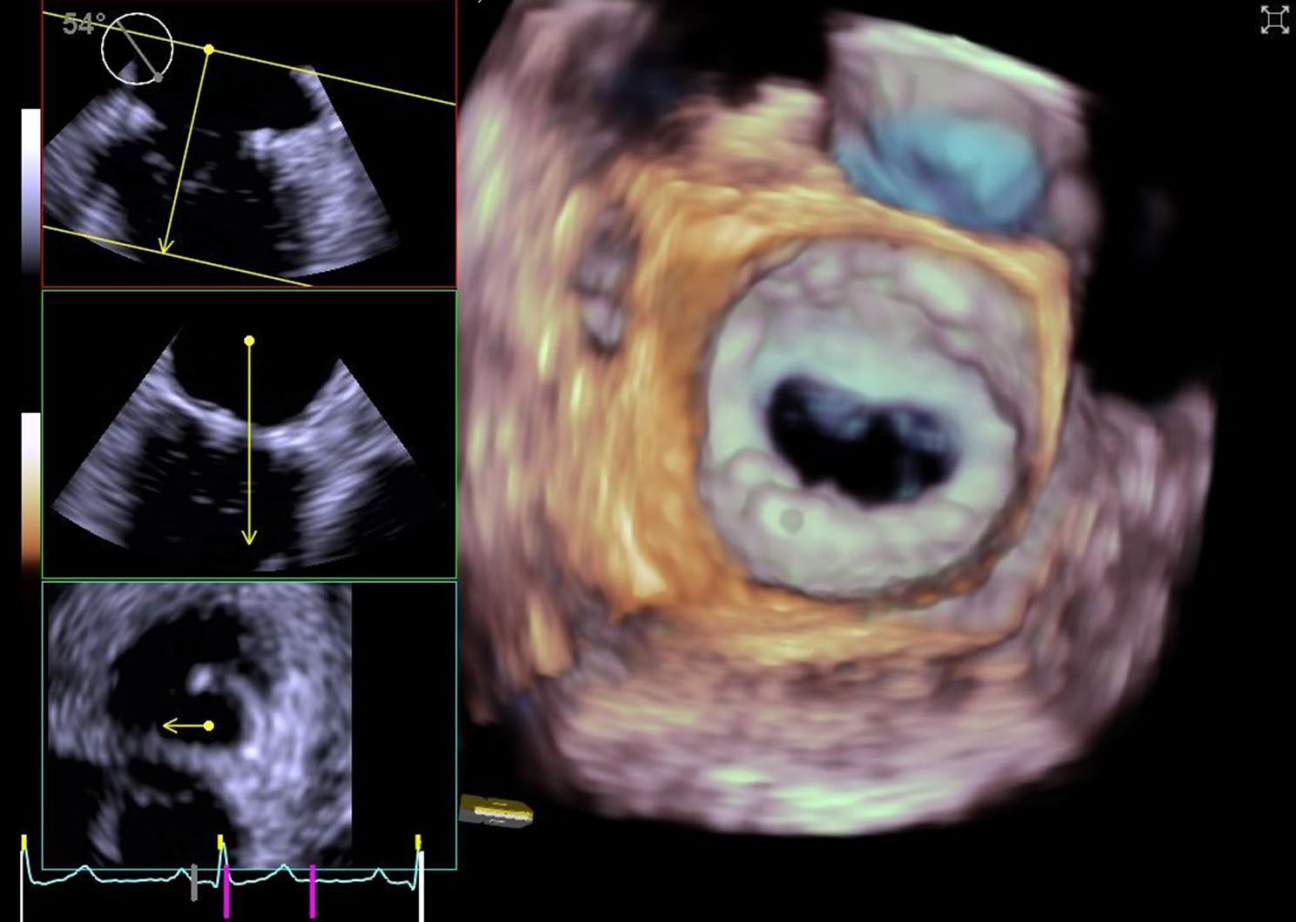
3D transesophagel examination of mitral valve (surgeon view) after the implant of the MEMO 4D annuloplasty ring

**Fig. 2:**
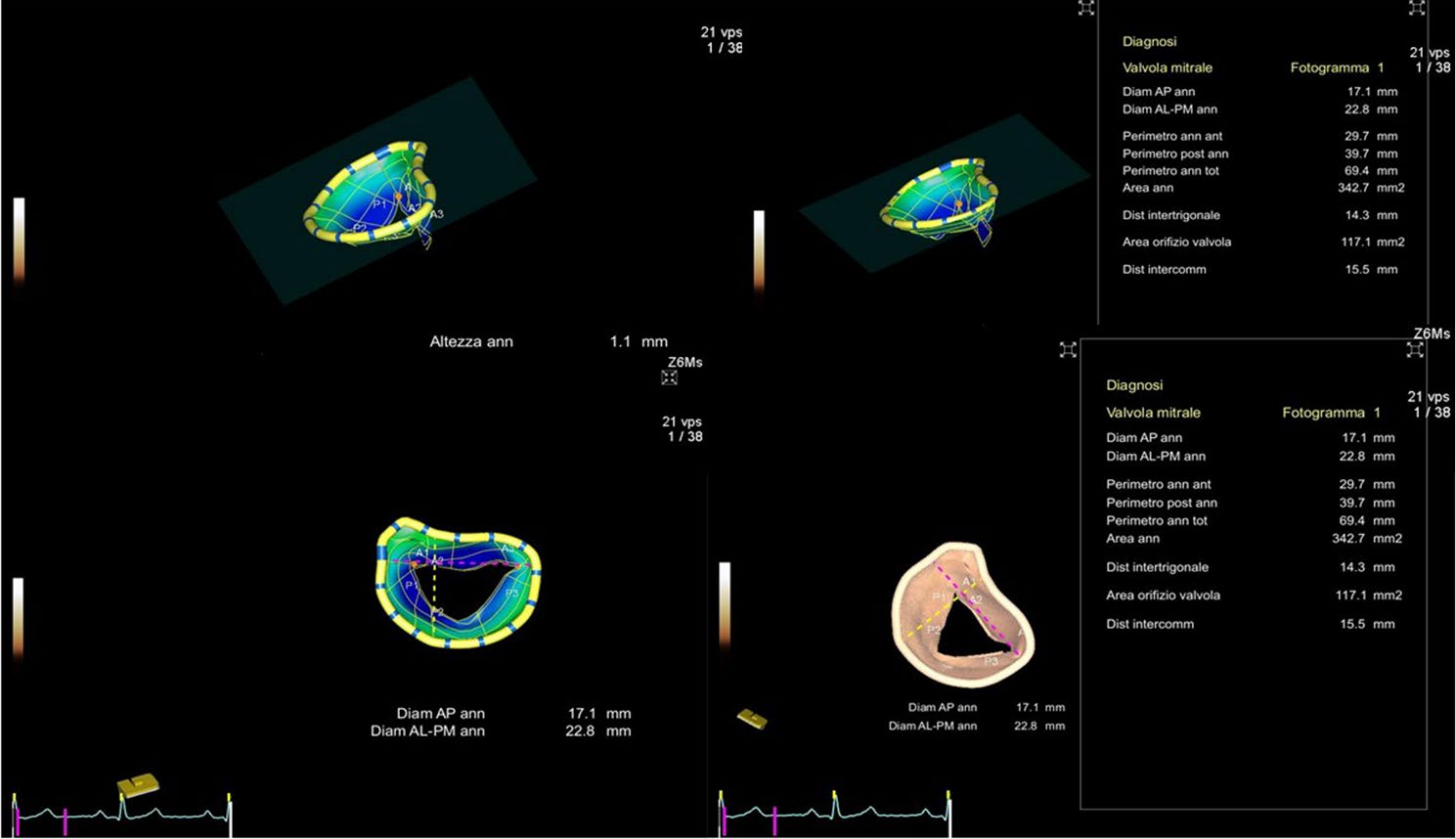
3-Dimensional echocardiographic reconstruction and assessment of mitral annulus with measurement of mitral annulus dynamics at end-systole and end-diastole

All patients who underwent mitral valve repair with Memo 4D ring at our clinical during the study period were contacted for a telephone follow-up within February 2022. No specific exclusion criteria were applied. Patients underwent clinical visit and transthoracic echocardiography at discharge from our clinic. During the telephone follow-up, they were asked to consent to undergo transesophageal echocardiography (TEE) at our clinic. 3D-TEE images of the mitral valve (Fig. 1) were acquired with a Siemens Acuson SC2000 (Siemens Medical Solutions USA, Inc., Issaquah, WA USA). The 3-D images were then analyzed with a post-processing software (Easyvalves) (Fig. 2). In the TEE examination for each patient, mitral annulus (MA) measurements included the intercommissural distance, the anteroposterior diameter, the height (distance in mm between the highest and the lowest insertion point of the MA in connection to the aortic annulus), the MA circumference and the MA area. The AHCWR was calculated as a parameter to reflect the saddle shape of the MA. To assess the MA dynamics during the cardiac cycle, the difference between the MA measurements at end-systole (ES) and end-diastole (ED) was expressed as the relative change in percentage.

### Annuloplasty device

The MEMO 4D semirigid ring contains an elastic nitinol and titanium alloy core that proved to accommodate the anatomic saddle shape of the mitral valve annulus and to restore the physiologic diameter ratio [9]. The core is enclosed in a sewing ring made of silicone and polyester fabric and covered a Carbofilm coating to address the annular motion restrictions related to endocardial ingrowth. The device exhibits selective flexibility, with maximum flexibility in the posterior portion of the ring and it has been reported to maintain physiological dynamics in the long term [10] and to show increased folding dynamics in comparison with other semi-rigid rings [11]. It features an anterior gradual saddle shaping and an increased antero-posterior dimension from size 34 to 42, to better accommodate the excess of leaflet tissue typically associated to the degenerative mitral diseases and allow its use in enlarged annuli, which lose contractility and the ability to saddle-shape in systole.

### Surgical procedure

The operation was performed according to the standard hospital practice. Central arterial and venous cannulation was done for all cases of combined procedures with full sternotomy access, while for isolated mitral repair surgeries a minimally invasive approach through right mini-thoracotomy was performed with peripheral femoral cannulation. Cold crystalloid cardioplegia was delivered in the aortic root in all cases. After the exposure and analysis of the mitral valve and restoration of the leaflets coaptation, the repair procedure was completed with the implant of a MEMO 4D ring. All the patients underwent intraoperative transesophageal echocardiographic assessment before and after weaning from cardiopulmonary bypass to assess transmitral gradients, and presence of residual regurgitation.

### Study endpoints

The primary endpoint of the study was to evaluate the safety of the MEMO 4D ring in the early postoperative period (up to discharge) through all cause, cardiac, and device related mortality and device related complications.

The secondary endpoint was to evaluate the performance of the MEMO 4D ring in the early postoperative period through improvement of hemodynamic post-implant assessed through the reduction of MR from baseline. Additional secondary endpoint was the assessment of the MA dynamics during the cardiac cycle.

### Statistics

Data were analyzed using descriptive statistics, with categorical variables presented as absolute values and frequencies (%) and the continuous variables presented as the mean and standard deviation (SD). All statistical analyses were performed using the software R, version 4.2.0 (R Core Team (2022). R: A language and environment for statistical computing. R Foundation for Statistical Computing, Vienna, Austria.).

## Results

A total of 53 patients were included in this study. The baseline characteristics of the patient cohort are summarized in Table 1. The majority of patients presented with degenerative mitral valve regurgitation (*N* = 44; 83%). Nine patients (17%) were classified as functional mitral regurgitation; 4 of them had an ischemic underlying pathology, while in the remaining 5 cases, the mitral regurgitation was caused by an isolated annulus dilatation, classified as functional Carpentier type I regurgitation. The grade of mitral regurgitation was equal or more than 3 + in 98.1% of the patients (*N* = 52). Mean patient age was 63.6 ± 11.7 years and associated cardiac diseases were present in 58.5% of the patients (*N* = 31).

**Table 1:** Patients characteristics

	n (%)
Age (years) (mean ± SD)	63.6 ± 11.7
Male	38 (71.7)
Degenerative MR	44 (83.0)
Functional MR	9 (17.0)
Associated cardiac disease	31 (58.5)
**NYHA class**	50 (94.3)
- Class I	3 (5.7)
- Class II	21 (39.6)
- Class III	29 (54.7)
**Cardiac rhythm**	
- Sinus rhythm	42 (79.2)
- Permanent atrial fibrillation	8 (15.1)
- Paroxysmal atrial fibrillation	2 (3.8)
- Paced	1 (1.9)
**MR grade**	
- 2+	1 (1.9)
- 3+	7 (13.2)
- 4+	45 (84.9)
LVEDD (mean ± SD) (mm)	56.7 ± 6.7
LVESD (mean ± SD) (mm)	40.4 ± 9.2
LVEF % (mean ± SD)	56.3 ± 10.6

*MR* mitral regurgitation, *LVEDD* left ventricular end diastolic diameter, *LVESD* left ventricular end systolic diameter

The surgical approach was full sternotomy in 54.7% (*N* = 29) of the cases in case of concomitant procedures; isolated mitral repair was performed with right mini-thoracotomy approach (*N* = 24, 45.3%). Anterior leaflet was treated in 13.2% of the cases (*N* = 7), while the posterior in 47.2% (*N* = 25) and both leaflets in 3 cases (5.7%). Neochords were used in 24 patients (45.3%). Triangular resection was performed in case of P2 prolapse, while in case of anterior leaflet prolapse a couple of artificial chordae were anchored on the prolapsed scallop.

The most common concomitant procedures were CABG in 24.5% (*N* = 13) and aortic valve replacement in 11.3% (*N* = 6) of the cases. Tricuspid annuloplasty was performed in 11 cases (20.8%). The most used ring size was size 34 mm (*N* = 30, 56.6%). Other details on operative data can be found in Table 2.

**Table 2:** Operative data

Operative data	N (%)
**Surgical approach**	
- Mini-thoracotomy	24 (45.3)
- full sternotomy	29 (54.7)
Anterior leaflet	7 (13.2)
Posterior leaflet	25 (47.2)
Both	3 (5.7)
Neochord use	24 (45.3)
**Number of neochord**	
1	10 (19.8)
2	13 (24.5)
3	1 (1.9)
**Concomitant procedures**	
- CABG	13 (24.5)
- Tricuspid surgery	11 (20.8)
- AVR	6 (11.3)
- Other	7 (13.2)
- Atrial fibrillation ablation	1 (1.9)
**Ring size**	
- 34	30 (56.6)
- 36	12 (22.6)
- 38	6 (11.3)
- 40	5 (9.4)
CPB time (min) (mean, SD)	106.9 (29.3)
Cross-clamp time (min) (mean, SD)	85.3 (22.9)

*CABG* coronary aortic bypass graft, *AVR* aortic valve replacement, *CPB* cardio-pulmonary bypass

There was no intraoperative or hospital mortality. No cases of stroke, bleeding, endocarditis or other major complications occurred. Postoperative results are reported in Table 3.

**Table 3:** Postoperative results

Postoperative results	N (%)
Hospital mortality	–
Reintervention	–
Stroke	–
**NYHA class**	
- I	38 (71.7)
- II	15 (28.3)
- III/IV	–
Bleeding	–
Acute myocardial infarction	–
Endocarditis	–
Thrombosis	–
ICU stay (days) (mean, SD)	2.8 (2.0)
Hospital stay (days) (mean, SD)	15.2 (6.7)

*ICU* intensive care unit, *SD* standard deviation

Mean intensive care unit and hospital stay were 2.8 and 15.2 days, respectively. At discharge, most patients were in NYHA class I.

Postoperative echocardiographic results (Table 4) showed no (90.6%) or 1+ (5.7%) mitral valve regurgitation. Only 1 patient (1.9%) presented with mitral valve regurgitation grade 2+. Mean postoperative transvalvular gradient was low (mean = 3.3 ± 1.2 mmHg). No cases of LVOT obstruction or systolic anterior motion occurred.

**Table 4:** Postoperative echocardiographic results

Postoperative echo results	N (%)
**Mitral regurgitation**	
- 0	49 (92.4)
- 1+	3 (5.7)
- 2+	1 (1.9)
- 3+	–
LVOT obstruction	–
SAM	–
Mean gradient (mmHg)	3.3 ± 1.2

*LVOT* left ventricular outflow tract, *SAM* systolic anterior motion

During follow-up, performed at a mean of 348.6 ± 260.2 days, two cases of re-admission due to congestive heart failure related to endocarditis occurred. In one case, the endocarditis was diagnosed on the aortic prosthesis and on the mitral and tricuspid valve; the patient died at 90 days postoperatively and the death was considered cardiac and device-related. In the second case, the patient was readmitted at the hospital at 47 months postoperatively and re-operated due to endocarditis.

No cases of thrombosis, dehiscence or other device related complications occurred.

The transesophageal echocardiographic follow-up was performed in a very limited number of patients (6) due to refusal of the TEE. The results of the mitral annulus dynamics are reported in Table 5. For all the parameters assessed, the values changed between systole and diastole according to the cardiac cycle physiological movements.

**Table 5:** Transesophageal echocardiographic results at follow-up

Parameter	Phase	Value	% change
IC diameter (mm)	Systole	26.3 ± 3.0	12.8%
	Diastole	30.1 ± 3.8	
AP diameter (mm)	Systole	20.5 ± 1.9	21.7%
	Diastole	26.2 ± 5.2	
Height (mm)	Systole	4.2 ± 7.3	2.0%
	Diastole	4.3 ± 2.5	
MA circumference (mm)	Systole	75.9 ± 10.8	19.0%
	Diastole	93.6 ± 17.6	
MA area (mm^2^)	Systole	411.3 ± 153.0	37.3%
	Diastole	656.0 ± 276.7	
AHCWR (%)	Systole	0.035 ± 0.043	77.2%
	Diastole	0.153 ± 0.086	

*IC* intercommissural, *AP* anterior-posterior, *MA* mitral annular, *AHCWR* annular height commissural width ratio

## Discussion

The primary objective of this study was to collect our early single center experience with the use of the semirigid mitral MEMO 4D ring. The preliminary results of this report show that mitral valve repair with the MEMO 4D ring was a reliable choice with very satisfactory postoperative outcomes. There were no in-hospital deaths, and a very limited number of postoperative complications occurred. The relatively long hospital stay (15.2 ± 6.7 days) was related to the lack of connection with a rehabilitation center; therefore, the hospital practice was to keep the patients longer in the hospital to allow them to return home with higher level of functional autonomy. At the mid-term follow-up, only 2 cases of endocarditis were reported (one case leading to death and one leading to reoperation), while no other device-related complications occurred.

Mitral insufficiency was well controlled, with most patients having either no or mild regurgitation at discharge. Mean transmitral gradients were low and aligned with previous experienced published on the MEMO 3D ring [7, 12], of which MEMO 4D represents an evolution. There were no LVOT obstruction or cases of systolic anterior motion.

The effectiveness of the MEMO 4D ring was also demonstrated by the improvement of patients’ clinical status (100% of patients in NYHA classes I and II).

All the patients included were treated with ring of sizes featuring the improved saddle-shape design and allowed to treat patients with larger and dilated annuli (in 9% of the patients a ring size 40 were used) with good postoperative outcomes, confirming the safety and effectiveness of the MEMO 4D ring.

The transesophageal echocardiographic follow-up, despite performed in a very limited number of patients, showed the restoration of the saddle shape of the mitral annulus while maintaining a physiological dynamic of the annulus (assessed as changes in AP diameter and AHCWR). Our results are aligned with the findings reported by Bouchez et al. [7], who compared with real-time 3D-TE, the mitral annular dynamics between MEMO 3D and Physio II rings, showing greater systolic–diastolic distensibility of the MEMO 3D, mimicking a dynamic saddle-shaped geometry through the cardiac cycle.

Our study presents several limitations. The first limitation is related to its retrospective single center nature, with all the implicit biases of such methodology. The other main limitations are the lack of a control group, the limited number of patients included and the paucity of transesophageal echocardiographic examination. Further multicentric prospective investigations on a more extensive number of patients are warranted also to determine the long-term outcomes of mitral repair with this semi-rigid annuloplasty ring. Additionally randomized controlled trials comparing the MEMO 4D with other annuloplasty rings will be needed to confirm the impact of the device characteristic on the functional outcome of the mitral valve repair. Some important parameters to assess the mitral valve repair outcomes, such as the coaptation height, tenting area, postero-lateral angle, left ventricular volumes and septal lateral distance, were not collected during the study. It was not feasible to perform a statistical test to assess the change of the mitral annulus dynamic parameters during the cardiac cycle due to the limited number of transesophageal echo data collected. Despite these limitations, our experience represents the first report on this relatively new semirigid annuloplasty ring.

## Conclusions

In conclusion, our series showed excellent mitral valve competency, reflected in the postoperative echocardiography results as well as the satisfactory early clinical outcomes. The echocardiographic follow-up, despite obtained in a limited number of patients, further confirmed the effectiveness of findings of this preliminary experience.

## Data Availability

Data are available from the corresponding author upon reasonable request.

## Abbreviations

AHCWR: annular height commissural width ratio
AP: anterior-posterior
CABG: Coronary artery bypass graft
CPB: Cardiopulmonary bypass
IC: intercommissural
ICU: intensive care unit
LVEDD: Left ventricular end-diastolic diameter
LVEF: Left ventricular ejection fraction
LVESD: Left ventricular end-systolic diameter
LVOT: left ventricular outflow tract
MA: mitral annular
MR: mitral regurgitation
MV: Mitral valve
NYHA: New York Heart Association
SAM: systolic anterior motion
SD: standard deviation
